# A Practical Treatise on Diseases of the Eye

**Published:** 1855-12

**Authors:** 


### BOOK NOTICES.

A Practical Treatise on Diseases of the Eye. By WILLIAM MACKENZIE, M. D., &c., &c., to which is prefixed an Anatomical Indtroduction Explanatory of a Horizontal section of the human Eyeball. By THOS. WHARTON JONES, F. R. S., &c., with one hundred and seventy-five Illustrations from the fourth and enlarged London edition, with notes and additions. By ADINELL HEWSON, A. M., M. D., &c. Philadelphia, Blanchard & Lea, 1855.

THE demand for a new edition of Dr. Mackenzie's very able work is a sufficient evidence that it is appreciated by the profession. In Europe it has been rendered into three of the continental languages, the French, German, and Italian. There are few professional authors whose works have met with such general favor.

For sale by Keen & Lea, Chicago, Ill. J.

